# Small RNAs in Seminal Plasma as Novel Biomarkers for Germ Cell Tumors

**DOI:** 10.3390/cancers13102346

**Published:** 2021-05-13

**Authors:** Nina Mørup, Rytis Stakaitis, Ieva Golubickaite, Meritxell Riera, Marlene Danner Dalgaard, Mikkel H. Schierup, Niels Jørgensen, Gedske Daugaard, Anders Juul, Kristian Almstrup

**Affiliations:** 1 Department of Growth and Reproduction, Copenhagen University Hospital-Rigshospitalet, 2100 Copenhagen, Denmark; nina.moerup.nygaard@regionh.dk (N.M.); rytis.stakaitis@lsmuni.lt (R.S.); ieva.golubickaite@lsmuni.lt (I.G.); Niels.Joergensen@regionh.dk (N.J.); Anders.Juul@regionh.dk (A.J.); 2 International Center for Research and Research Training in Endocrine Disruption of Male Reproduction and Child Health (EDMaRC), Copenhagen University Hospital, 2100 Copenhagen, Denmark; 3 Laboratory of Molecular Neurooncology, Lithuanian University of Health Sciences, LT-50161 Kaunas, Lithuania; 4 Department of Genetics and Molecular Medicine, Lithuanian University of Health Sciences, LT-44307 Kaunas, Lithuania; 5 Bioinformatics Research Centre, Aarhus University, 8000 Aarhus C, Denmark; mtxellrb@gmail.com (M.R.); mheide@birc.au.dk (M.H.S.); 6 DTU Multi-Assay Core, Department of Health Technology, Technical University of Denmark, 2800 Lyngby, Denmark; marld@dtu.dk; 7 Department of Oncology, Copenhagen University Hospital, 2100 Copenhagen, Denmark; kirsten.gedske.daugaard@regionh.dk; 8 Department of Clinical Medicine, University of Copenhagen, 2100 Copenhagen, Denmark

**Keywords:** small RNAs, testicular cancer, diagnostics

## Abstract

**Simple Summary:**

Testicular cancer is the most common cancer among young men. It is rarely diagnosed at early stages, being only detected with a highly invasive procedure that presents notable side-effects. Circulating small RNAs have recently been identified as testicular tumor markers, but are unable to diagnose testicular cancer at an early pre-invasive stage. So far, studies have been limited to microRNAs, with other small RNAs remaining unexplored as likely biomarkers. By sequencing all small RNAs in semen samples from men with different stages of testicular cancer and healthy men, we identify signatures predictive of cancer, even at an early stage. Thus, our study provides great potential for non-invasive early diagnosis of testicular cancer. Extensive biological variance in small RNA levels across samples, together with small sample sizes, limit the power to detect single small RNA markers. Hence, larger studies are needed to confirm our findings and deduce their full diagnostic capacity.

**Abstract:**

Circulating miRNAs secreted by testicular germ cell tumors (TGCT) show great potential as novel non-invasive biomarkers for diagnosis of TGCT. Seminal plasma (SP) represents a biofluid closer to the primary site. Here, we investigate whether small RNAs in SP can be used to diagnose men with TGCTs or the precursor lesions, germ cell neoplasia in situ (GCNIS). Small RNAs isolated from SP from men with TGCTs (*n* = 18), GCNIS-only (*n* = 5), and controls (*n* = 25) were sequenced. SP from men with TGCT/GCNIS (*n* = 37) and controls (*n* = 22) were used for validation by RT-qPCR. In general, piRNAs were found at lower levels in SP from men with TGCTs. Ten small RNAs were found at significantly (q-value < 0.05) different levels in SP from men with TGCT/GCNIS than controls. Random forests classification identified sets of small RNAs that could detect either TGCT/GCNIS or GCNIS-only with an area under the curve of 0.98 and 1 in ROC analyses, respectively. RT-qPCR validated hsa-miR-6782-5p to be present at 2.3-fold lower levels (*p* = 0.02) in the SP from men with TGCTs compared with controls. Small RNAs in SP show potential as novel biomarkers for diagnosing men with TGCT/GCNIS but validation in larger cohorts is needed.

## 1. Introduction

Type II testicular germ cell tumors (TGCT) comprise a heterogeneous group of neoplasms that mainly affect young and adolescent men (median age 32 years). There are two types of TGCTs, homogeneous seminomas and heterogeneous non-seminomas, which can consist of varying combinations and proportions of embryonal carcinoma, yolk sac tumor, choriocarcinoma, and teratoma. It is believed that TGCTs originate from fetal germ cells (primordial germ cells or gonocytes) that arrest during development [1]. If arrested fetal germ cells are not eradicated during development, they are thought to transform into TGCT precursor cells named germ cell neoplasia in situ (GCNIS). GCNIS shares many similarities with fetal germ cells [2,3,4] and detecting GCNIS is possible before it becomes invasive and manifests as a TGCT. However, GCNIS is rarely diagnosed since the lesion is asymptomatic. Diagnosis of GCNIS most often occurs in patients at increased risk of developing a TGCT and can only be firmly established using immunohistochemical staining of the testicular biopsy for the presence of, e.g., placental-like alkaline phosphatase (PLAP) [5]. Among men with a unilateral TGCT, GCNIS is found in approximately 5% of the contralateral testes [6], and in some countries like Denmark and Germany, a contralateral testicular biopsy is performed together with orchiectomy of the affected testis [1]. A negative contralateral biopsy reduces the risk of a metachronous TGCT albeit failure of routine histological GCNIS detection has been reported [7]. However, the indication to perform a contralateral biopsy remains controversial. A contralateral biopsy may result in earlier diagnosis with the potential of early treatment and limiting treatment-induced side effects but it also represents an invasive procedure with the risk of developing edema, superficial hematomas, or infections, especially in cases with small testes [6]. Hence, there is a great need for non-invasive methods to diagnose GCNIS to avoid side-effects from the invasive procedure.

Several studies have reported on non-invasive methods to diagnose GCNIS and most have focused on the ejaculate as a diagnostic media [8]. GCNIS cells have been identified in the ejaculate [9], where they are occasionally exfoliated, and assays using staining of OCT3/4 [10] or PLAP and TFAP2C together [11,12] have been developed to identify exfoliated GCNIS. However, the sensitivity of these assays remained low, most likely because GCNIS detection was limited by the formation of a solid tumor that eventually could block exfoliation. More recently, a panel of microRNAs (miRNA) has gained increasing interest as novel biomarkers for TGCTs [13] and their usefulness as biomarkers for GCNIS has also been investigated. First, the levels of miR-371a-3p and miR-367-3p were investigated in serum from patients with GCNIS, but only 52% of the patients showed elevated miRNA levels [14]. Since GCNIS was only occasionally detected by miR-371a-3p in serum [14] and serum miR-371a-3p levels correlate with tumor size [15], likely, a certain number of GCNIS cells is needed for proper miR-371-3p detection in serum. This is supported by studies showing that the level of miR-371a-3p in men with a TGCT is higher in blood from the testicular vein than the cubital vein [16,17,18]. Thus, it is likely that levels of biomarkers present in body fluids close to the primary site are more informative than circulating levels. This is also evidenced by increased sensitivity of detecting intracranial childhood germ cell tumors by analyzing miRNAs in cerebrospinal fluid rather than serum [19]. The level of miR-371a-3p was also measured in seminal plasma (SP) from patients with TGCTs. However, miR-371a-3p levels were not elevated in patients with TGCTs but instead correlated with sperm concentration and total sperm count [20]. Thus, neither serum nor SP levels of miR-371a-3p can be used to diagnose GCNIS. Investigations of SP have so far been limited to miRNAs and not to other types of small RNAs. Hence, other types of small RNAs present in SP could be more sensitive than miRNAs in diagnosing GCNIS and TGCTs. Furthermore, it is currently a biological puzzle why some testicular-derived small RNAs are present in circulation and not SP and vice versa. Here, we use small RNA sequencing to investigate which small RNAs are found in the SP from men with TGCTs and GCNIS and explore their potential as diagnostic biomarkers for TGCT and GCNIS.

## 2. Materials and Methods

### 2.1. Study Subjects

The study population consisted of patients with GCNIS, TGCT, and five control groups as outlined in Table 1. SP samples from patients with GCNIS and TGCT (divided into two groups with either seminoma or non-seminoma) were all obtained when the men were delivering a semen sample for cryopreservation before treatment. The sperm concentrations were, however, blinded to us. At the time of sample collection, the patient diagnoses were unknown, therefore, we also collected samples from men with other lesions than TGCT/GCNIS (specified in Table 1), which served as a cryopreservation control group. Because the small RNAs present in SP could be affected by the degree of ongoing spermatogenesis, we included three control groups with different sperm concentrations. This included a group with low sperm concentrations (<15 million/mL), a group with medium sperm concentrations (>20 and <80 million/mL) and a group with high sperm concentrations (>100 million/mL). Finally, a small group of semen donors was included.

Patients with GCNIS, TGCTs, and cryopreservation controls were recruited from 2016 to 2017 when they visited the semen bank at the Department of Growth and Reproduction (Copenhagen University Hospital) for cryopreservation. The patients delivered a semen sample for cryopreservation and if the sperm concentration was high enough for sufficient sperm to be cryopreserved, 200 µL of the sample was aliquoted into an Eppendorf tube and the SP isolated and frozen for the current study (see below).

Patients with low, medium, and high sperm concentrations were recruited for the study from 2018 to 2019 when they attended our semen laboratory for andrological work-up. After assessment of semen quality, the remaining sample was transferred to Eppendorf tubes and SP was isolated and frozen (see below). The semen donors were included from internal quality control programs in the semen laboratory during 2017–2018 and the SP was isolated and stored similarly to all other samples.

An independent validation cohort was gathered to validate the sequencing results by RT-qPCR. This consisted of SP from men with non-seminomas (*n* = 18), seminoma (*n* = 17), GCNIS (*n* = 3) (Table S1), and cryopreservation controls (*n* = 25). No patients overlapped between the sequencing cohort and the validation cohort. After technical quality evaluation, 4 samples (3 controls and 1 GCNIS) were excluded from the validation analysis due to inefficient RNA isolation or cDNA synthesis according to the spike-in measurements.

### 2.2. Isolation of Seminal Plasma

The semen samples were left to liquefy, and seminal plasma was isolated between 30 min (for SP from patients with GCNIS or TGCT and cryopreservation controls) and 5 h (for SP from low, medium and high sperm concentration control groups and semen donors) after delivery. Aliquots of 200 µL were centrifuged for 15 min at 800× *g*, the supernatant transferred to new tubes, which were centrifuged again for 10 min at 16,000× *g* and the supernatant transferred to new tubes and stored at −80 °C.

### 2.3. RNA Isolation from Seminal Plasma

RNA was isolated using Trizol LS (TRI) reagent (Invitrogen, Carlsbad, CA, USA, cat. #: 10296028) according to the manufacturer’s instructions with minor adjustments. In brief, 100 µL SP and 300 µL TRI Reagent^®^ were mixed and incubated for 5 min. Then, 80 µL chloroform was added, mixed, and incubated for 2–3 min. The sample was centrifuged at 12,000× *g* at 4 °C for 15 min. The upper colorless phase was transferred to a new tube where 200 µL isopropanol was added, mixed, and incubated for 10 min. The sample was then centrifuged at 12,000× *g* at 4 °C for 10 min and the supernatant removed. The pellet was washed with 400 µL 75% ethanol, vortexed and centrifuged at 7500× *g* at 4 °C for 5 min. The supernatant was discarded, and the sample was vacuum-dried. The RNA pellet was resuspended in 20 µL RNase-free water, incubated on a heat block at 58 °C for 15 min and stored at −80 °C.

The quality of the isolated RNA was checked with the Agilent small RNA Bioanalyzer kit (Agilent, Santa Clara, CA, USA) according to manufacturer’s instructions and RNA content was evaluated with the Qubit^®^ RNA High Sensitivity kit (Thermo Fisher Scientific, Waltham, MA, USA) using 2–5 µL sample.

### 2.4. Sequencing Library Preparation

Sequencing libraries were prepared using the CATS small RNA library kit (Diagenode, Seraing, Belgium) according to manufacturer’s instructions using 10 ng RNA and running 15 cycles of pre-amplification. The libraries were cleaned up using Agencourt^®^ AMPure^®^ XP beads (Beckman Coulter, Brea, CA, USA) according to manufacturer’s instructions, eluting the libraries in Qiagen Elution Buffer (Qiagen, Hilden, Germany).

The quality of the libraries was assessed on a Bioanalyzer using the Agilent DNA High Sensitivity kit and chips (Agilent) and the cDNA quantity was assessed using the Qubit DNA Broad Range kit (Thermo Fisher Scientific).

The libraries were pooled in two pools each containing 24 samples with different barcodes (100 ng in total) as outlined in Table 1. Sequencing was performed on an Illumina HiSeq 4000 with 150 bp paired-end reads (Illumina, San Diego, CA, USA).

### 2.5. Small RNA Alignment and Annotation

Fastq files were checked with FASTQC for quality, and CutAdapt [21] was used to trim the reads according to the instructions in the CATS small RNA library kit. The Oasis pipeline version 2.0 [22] was used to align trimmed reads of 15–50 bp in length to the following databases: Mirbase ver. 21, piRNAbank V.2 and Ensembl v84 (for snRNA, snoRNA and rRNA). Oasis uses STAR in a non-splice-junction-aware mode for alignment and we allowed 5% mismatches. All reads mapping to the above human databases were combined into one count matrix and analyzed in R (see below). Reads that did not map to human small RNAs were subsequently aligned against the reference genome (hg 38), to predict novel miRNAs with miRDeep2. We did not detect any novel miRNAs in any of the samples. Further details can be found in Rahman et al., 2018.

### 2.6. Analysis of Read-Counts of Human Small RNAs

The read-count matrix of human small RNAs was analyzed in R v. 3.6.1 using the edgeR package (v. 3.28.1) [23,24]. In total, 49,970 human small RNAs were identified. Small RNAs with less than 1 count per million in two samples (*n* = 29530) were filtered out, leaving 20,440 small RNAs for analysis. Small RNA reads were normalized to the library size and potential outliers were identified by inspection of multidimensional scaling (MDS) plots. Three samples, a seminoma (S2), a non-seminoma (NS8), and a control (H5) with high sperm concentration, were found to group distant from all other samples and were excluded from downstream analyses. The R package ggpubr (v. 0.2.5) [25] was used to draw violin plots and statistical differences were tested with a Wilcoxon non-parametric rank-sum test. Generalized linear models were used to identify differentially expressed small RNAs. Four different contrasts were considered: TGCT and GCNIS vs. controls, GCNIS-only vs. controls, seminoma vs. non-seminoma, and low vs. high sperm concentration. A false discovery rate (FDR) q-value of 0.05 was used as cut-off in the identification of differentially present small RNAs. The R package EnhancedVolcano (v. 1.4.0) [26] was used to draw volcano plots of the contrasts.

### 2.7. Random Forests Classification

Random forests classification [27] is a machine learning algorithm that tests many models of decision trees with random subsets of the input data and subsequently uses the combined result (the forest) for prediction. For a detailed description of random forests see Cutler et al., 2007 [28]. The random forests pipeline available in Oasis [22] was used for prediction. The sample size was balanced and the MTRY parameter optimized by the pipeline. The number of trees was set at 100,000 allowing an ample number of trees for classification. The out-of-bag (OOB) error obtained for each independent tree that forms the forest was minimal in all cases after 10,000 trees. Backwards feature pruning and 10-fold cross-validation were used to select the most informative small RNAs. Based on the random forests classifications, receiver operating characteristics (ROC) curves were plotted, and the sensitivity and specificity calculated.

### 2.8. qPCR Quantification

RNA isolation from SP was performed as above, except that 1 µL of synthetic spike-in (0.5 ng/µL, ath-miR-159a, 5′Phos. TAG Copenhagen, Copenhagen, Denmark) was added to the TRI reagent to ensure technical quality control. The quantity of the isolated RNA was measured using a Qubit 3 fluorometer (Thermo Fisher Scientific) with the RNA HS assay kit (Thermo Fisher Scientific) and stored at −80 °C.

cDNA was synthesized using the reverse transcription (RT) primer pool from the custom TaqMan Small RNA Assays (Thermo Fisher Scientific) targeting six different small RNAs (four small RNAs identified by sequencing and two small RNAs used as controls) (Table S2). In brief, 2.5 µL of 20× individual RT primers were pooled and diluted to a final concentration of 0.05× of each primer. 3 µL of RNA (1–350 ng) was mixed with RT reaction mix, which contained 6 µL of RT primer pool, 0.30 µL of dNTPs (100 mM), 3 µL of MultiScribe Reverse Transcriptase (50 U/µL), 1.50 µL RT Buffer, 0.19 µL of RNase Inhibitor (20 U/µL), and 1.01 µL of Nuclease-free water. The total volume was 15 µL. After mixing, the plate was incubated on ice for 5 min, at 16 °C for 30 min, at 42 °C for 30 min, and at 85 °C for 5 min.

The preamplification reaction was performed with 5 µL of RT product, 12.5 µL of TaqMan Fast Advanced Master Mix (2×) (Thermo Fisher Scientific), 3.75 µL PreAmp primer pool and 3.75 µL of nuclease-free water. After mixing, the plate was incubated at 95 °C for 2 min, for 12 cycles at 95 °C for 15 s and at 60 °C for 1 min, and then held at 99.9 °C for 10 min. To dilute the product, 35 µL of 0.1× TE (pH 8.0) was added to 5 µL of each pre-amplified cDNA.

qPCR was performed on a QuantStudio 3 (Thermo Fisher Scientific) with the stan-dard cycling program (95 °C for 2 min, 50 cycles at 95 °C for 15 s, followed by 60 °C for 60 s), using 2 µL of cDNA, 1 µL of TaqMan probes (20×), and 10 µL of TaqMan Fast Advanced Master Mix (2×). The final volume of 20 µL was reached by adding nuclease-free water.

hsa-miR-6833-5p was selected as housekeeping small RNA after analysis of the small RNA sequencing results, where it was the most stably expressed small RNA in the cohort and it was used for ΔCt calculations. The difference between patient groups was evaluated using independent t-tests including Bonferroni correction by Python’s Stattannot package (version 0.2.3) on JupyterLab (version 2.1.5).

## 3. Results

### 3.1. Small RNAs Differentially Present in Seminal Plasma

**Figure 1:**
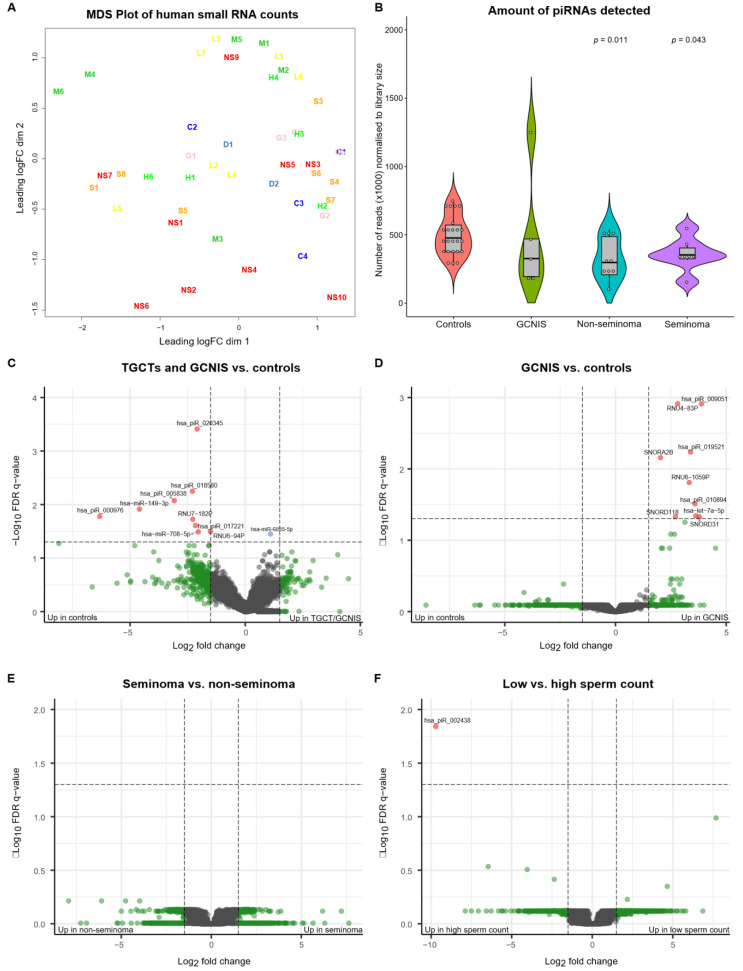
Multidimensional scaling (MDS) plot of the data, violin plot of the number of piRNA reads and volcano plots of indicated contrasts in the data. (**A**) MDS plot of all samples after exclusion of three outliers and normalization of the data to library size. No grouping of samples is obvious from the plot. Sample groups are further explained in Table 1. (**B**) Violin plot showing the number of piRNA-reads normalized to library size. A significantly larger number of piRNA-reads is obtained from seminal plasma from controls compared with men with non-seminoma (*p*-value: 0.011) and seminoma (*p*-value: 0.043). A similar but insignificant difference is observed when men with GCNIS-only are compared with controls. The indicated *p*-values are based on a non-parametric rank-sum test (Wilcoxon) with controls as reference. (**C**) Volcano plot of small RNAs (*n* = 20,440), indicating small RNAs differentially present in seminal plasma from men with a TGCT or GCNIS and controls. Five out of the 10 small RNAs showing significant differences are piRNAs present at higher levels among controls. Red dots indicate small RNAs that show >1.5-fold difference and with an FDR *q*-value < 0.05. Blue dots are significant but at fold difference < 1.5 and green dots are not significant but show >1.5-fold difference. (**D**) Volcano plot of GCNIS vs. controls, indicating that all small RNAs that show significant differences appear at higher levels in the seminal plasma from men with GCNIS and represent all types of small RNAs. (**E**) Volcano plot of seminoma vs. non-seminoma indicating no significant differences. (**F**) Volcano plot of low vs. high sperm concentrations, indicating only a single piRNA (has-piR-002438) was found at higher levels among men with high sperm concentrations. Abbreviations; C: cryopreservation control, D: donor, G: GCNIS, H: high sperm concentration control, L: low sperm concentration control, M: medium sperm concentration control, NS: non-seminoma, and S: seminoma.

As outlined in Table 1, 48 samples were included in the sequencing cohort, representing 23 men with either TGCT (*n* = 18; 8 seminoma and 10 non-seminoma) or GCNIS-only (*n* = 5) and 25 controls without a TGCT. Inspection of the multidimensional scaling (MDS) plot revealed three samples (a seminoma (S2), a non-seminoma (NS8) and a control with high sperm concentration (H5)) that appeared to be outliers (Figure S1), and these were removed from subsequent analysis. After removing the outliers, the MDS plot revealed an even distribution of all samples with no specific grouping of samples (Figure 1A). Analysis of the number of reads (normalized to the library size) according to the type of small RNA revealed that miRNAs were the most abundant type of small RNA detected and that significantly fewer piRNA-reads were detected in samples originating from men with a TGCT compared with controls (*p* = 0.011 for seminoma and *p* = 0.043 for non-seminoma) (Figure 1B, Figure S2). A similar trend was observed for men with GCNIS albeit this difference was insignificant (Figure 1B). None of the other types of small RNAs revealed significant differences (Figure S2).

We tested whether any small RNAs were differentially present in SP from men with either a TGCT or GCNIS compared with controls. Using an FDR cut-off q-value of 0.05, we identified nine small RNAs that appeared at significantly higher levels in the SP from controls than in SP from men with either a TGCT or GCNIS. Only one small RNA was found at higher levels in SP from men with TGCT/GCNIS (Table 2, Figure 1C). Nine small RNAs were found at higher levels in SP from men with GCNIS-only compared with controls (Table 2, Figure 1D). No small RNAs were found to be differentially present in SP according to the type of TGCT (Figure 1E) and only one small RNA was found at higher levels in SP from men with high sperm concentrations compared with men with low sperm concentrations (Table 2, Figure 1F).

### 3.2. Random Forests Classification

**Figure 2:**
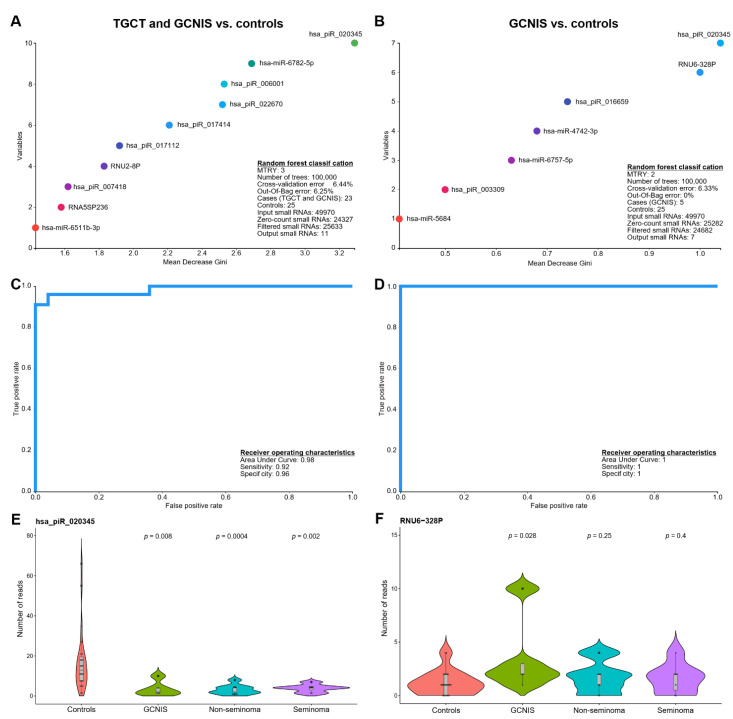
Application of machine learning to classify seminal plasma small RNAs according to whether a solid TGCT or GCNIS was present. Random forests classification was performed with a sample size balanced setup and with the specified parameters, MTRY and number of trees, optimized for the setup. A backwards feature pruning and 10-fold cross-validation were used to select the most informative small RNAs. The plot shows the top-10 most informative small RNAs determining the classification rule for (**A**) TGCT/GCNIS vs. controls and (**B**) GCNIS vs. controls. The cross-validation and the out-of-bag error indicate the performance of the classification. Based on the random forests classifications receiver operating characteristics (ROC) curves were drawn and the sensitivity and specificity calculated for (**C**) TGCT/GCNIS vs. controls and (**D**) GCNIS vs. controls. (**E**,**F**) shows violin plots of two small RNAs that were among the most informative in the classification. The indicated *p*-values are based on a non-parametric rank-sum test (Wilcoxon) with controls as reference.

To deduce whether a combination of the small RNAs could be used to classify men according to whether or not they had a TGCT or GCNIS, we applied the machine learning technique random forests classification. Using a 10-fold cross validation, the random forests classification identified 11 small RNAs that were predictive of whether a TGCT or GCNIS was present (Figure 2A, Table 3) and seven small RNAs predictive of whether GCNIS-only was present (Figure 2B, Table 3). These sets of small RNAs were subsequently used to determine the diagnostic performance using receiver operating characteristics (ROC) curves and revealed an area under the curve (AUC) of 0.98, a sensitivity of 0.92 and a specificity of 0.96 for predicting whether a TGCT or GCNIS was present (Figure 2C). For GCNIS-only the AUC was 1 (Figure 2D). It is important to note that the GCNIS-only group is substantially smaller (*n* = 5) than the combined group of TGCT and GCNIS (*n* = 23), which makes random forests prediction less useful. Overall, six of the 11 small RNAs (55%) identified in the random forests classification of TGCT/GCNIS were piRNAs (Table 3). Only three out of seven small RNAs (43%) in the GCNIS-only group were piRNAs (Table 3), which could indicate a more pronounced loss of germ cell-specific piRNAs in men with TGCTs. Interestingly, the most informative small RNA was in both cases hsa_piR_020345, which was found at higher levels among controls (Figure 2E). However, snRNAs like RNU-328P were found at significantly higher levels (*p*-value: 0.028) in SP from men with GCNIS-only (Figure 2F).

### 3.3. Validation of Sequencing Results

**Figure 3:**
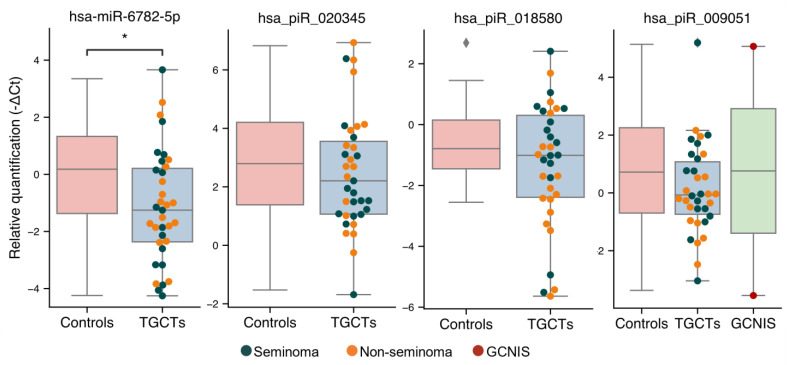
Validation of small RNA levels in SP by RT-qPCR. Levels of hsa-miR-6782-5p, hsa_piR_020345, hsa_piR_018580, and hsa_piR_009051 in SP from the validation cohort of men with TGCTs (*n* = 37) and controls (*n* = 22) were assessed by RT-qPCR. Expression data are shown as -∆Ct values normalized to hsa-miR-6833-5p, which was found to be the stable across all samples in the sequencing analysis and imply that a lower value indicates a lower level of the small RNA in SP. Differently colored dots represent patients with seminoma (blue), non-seminoma (orange) and GCNIS (red). * indicates a *p*-value < 0.05.

From the above analyses, four small RNAs (hsa-miR-6782-5p, hsa_piR_020345, hsa_piR_009051, and hsa_piR_018580) were selected for further investigation of their usefulness in distinguishing men with TGCTs from controls. In accordance with the sequencing results, hsa-miR-6782-5p was present at significantly lower levels in the SP from men with TGCTs from the validation cohort compared with controls (mean difference of 1.19 qPCR’s cycles or 2.28-fold decrease, *p* = 0.02; Figure 3, Figure S3). Additionally, a decrease in both hsa_piR_018580 and hsa_piR_020345 levels were detected in the SP from TGCT patients of the validation cohort (Figure 3), albeit the differences did not reach statistical significance. Including the two GCNIS-only samples from the validation cohort did not change the results. Finally, hsa_piR_009051, which was present at higher levels in men with GCNIS in the sequencing cohort, could not be validated with only two GCNIS-only samples in the validation cohort. No difference was observed for hsa_piR_009051 between the control and TGCT groups (Figure 3).

Generally, all piRNAs selected for validation showed a trend of decreased quantities in TGCT patients, which was also observed in the sequencing cohort.

## 4. Discussion

This is the first study to quantify many different types of small RNAs in seminal plasma, which separate men with GCNIS and TGCTs from controls. We were able to predict most cases and controls into the right categories by applying a machine learning technique on the sequencing data and obtain a nearly perfect diagnostic performance. However, our data also reveal a great overlap between controls and men with TGCTs in the measured levels of specific small RNAs (Figure S3). Only one of the small RNAs (hsa-miR-6782-5p) was confirmed to be present at significantly lower levels in SP from men with TGCTs compared with controls in the validation cohort. Nevertheless, the mean levels of both hsa_piR_018580 and hsa_piR_020345 showed a non-significant shift in the expected direction. Hence, in order to use small RNAs in SP to diagnose TGCT or GCNIS, several small RNAs should be measured, which limits its routine clinical use. The clinical use of this panel of small RNAs from SP is also limited since diagnosis of TGCTs by circulating miRNAs already show great diagnostic performance. However, because a non-invasive test for GCNIS diagnosis is greatly needed, measurement of several small RNAs in SP could be feasible for this patient group. Currently, GCNIS-only is rarely diagnosed, and it is a challenge to obtain a large enough sample size of SP from GCNIS-only patients to properly validate the diagnostic performance of the small RNAs. Furthermore, the GCNIS-only group could include a varying number of GCNIS cells and different degrees of ongoing spermatogenesis, and, therefore, the GCNIS-only group is expected to show a great degree of biological variability. This is exemplified by one of the samples from the GCNIS group from the sequencing cohort showing higher piRNA levels than the other GCNIS samples, potentially indicating more ongoing spermatogenesis in this patient than in the others (piRNAs are germ cell-specific). The AUC of 1 for prediction of GCNIS-only is probably also exaggerated and affected by the small sample size, especially because random forests classification works more efficiently with larger sample sizes.

Since TGCTs derive from GCNIS cells, patients with TGCTs most often have GCNIS cells adjacent to their tumors. Hence, theoretically, new diagnostic biomarkers for GCNIS should also be detectable in patients with TGCTs. However, we found no overlap between the small RNAs present at significantly different levels in the combined TGCT/GCNIS and the GCNIS-only groups. One piRNA (piR-020345) overlapped between the two random forests classification analyses of TGCT/GCNIS vs. controls and GCNIS-only vs. controls but it did not reach statistical significance in the validation cohort. This could be due to the TGCT cells having different expression profiles depending on the subtype and the specific composition of cancer cells found in the tumors. In addition to this, the level of ongoing spermatogenesis, the number of GCNIS-containing tubules and the size of the TGCT varies substantially from patient to patient. Since piRNAs are germ cell specific, the levels of piRNAs are probably highly associated with the number of functioning germ cells and the level of ongoing spermatogenesis. It may be difficult to distinguish changes in piRNA levels due to the level of ongoing spermatogenesis from changes caused by the presence of GCNIS. However, only one piRNA (hsa-piR-002438) was identified to be present at significantly higher levels in men with high sperm concentrations compared with men with low sperm concentrations, contradicting ongoing spermatogenesis as the major factor affecting piRNA levels in SP. The difference in sperm concentration as well as the read-depth of the sequencing and the sample volume may not be sufficient to reveal significant changes in small RNA levels.

The observed biological variability of small RNA levels in SP is probably caused by variation in the contribution of several vesicles (e.g., seminal vesicles and prostate) to both the volume and the pool of small RNAs. According to SpermBase [29], only a few of the identified small RNAs are found in ejaculated sperm and most of the biological variation in our data probably originates from somatic and pre-meiotic cells. We did try to accommodate some of the biological variation by including men with different sperm concentrations as controls, but the variation was still considerable.

Except for hsa_piR_020345, none of the small RNAs identified to be differentially present in SP overlapped with the panel of informative small RNAs from the random forests classification. This is somewhat expected because machine learning classification takes advantage of other and combined features than simple statistical significance. This is the very essence of machine learning. Interestingly, hsa_piR_020345 was found to be predictive for both the combined TGCT/GCNIS group and the GCNIS-only group in the random forests prediction. hsa_piR_020345 (piRNAbank) is also known as piR-hsa-28160 (PiRBase), hsa-piR-28033 (piRNAdb) or DQ597945.1/piR-36011 (NCBI) and has been identified in the human testis before [30] but no potential mRNA targets are described in piRBase. Although piRNAs have been described to play functional roles in many cancers [31] including TGCTs [32,33] the fact that piRNAs, in general, appeared at lower levels in the SP from men with TGCTs most likely reflect that germ cells are found in lower numbers when a TGCT is present in the testis.

It would be interesting to analyze matching serum and SP samples to deduce whether there is any overlap in the small RNAs detected in serum and SP. We have previously identified piRNAs in circulation that correlated with reproductive hormones but hsa_piR_020345 was not found in circulation [34]. Together with the observation that miR-371a-3p is found in SP from non-TGCT men but not in circulation [35] this could indicate that only a selected subset of small RNAs is sequestered to the bloodstream, while another subset is sequestered to the SP. Previous studies evaluating small RNAs in SP have primarily focused on miRNAs [20,36,37] but a few studies have also looked at other types of small RNAs [38,39]. In addition to miRNAs, Vojtech et al., investigated which small RNAs were present in seminal exosomes and found a variety of small RNAs including tRNA fragments, and piRNAs [39]. One could speculate whether a specific set of exosomes are secreted into circulation and SP, respectively. In any case, a larger cohort of samples is needed to validate our findings and the diagnostic potential of small RNAs in SP.

## 5. Conclusions

Sets of small RNAs in seminal plasma show potential as novel biomarkers for diagnosing men with testicular germ cell cancer both at the invasive and pre-invasive stage. On its own, only hsa-miR-6782-5p was validated to discriminate men with TGCTs from controls. Larger confirmative studies are needed to verify the diagnostic performance of the identified small RNAs in seminal plasma.

## Figures and Tables

**Table 1:** Seminal plasma samples subjected to small RNA sequencing.

Sample ID	Group	Specification	Pool No.	CATS INDEX	Library Size after Trimming
C1	Cryo. control	Leydig cell tumor	1	1	10,179,217
C2	Cryo. control	TC post-orchiectomy	1	10	10,450,156
C3	Cryo. control	Extragonadal GCT	2	3	6,312,806
C4	Cryo. control	Epidermoid cyst	2	11	6,248,375
D1	Donor	Donor	1	3	8,279,605
D2	Donor	Donor	2	2	4,786,872
L1	Low sperm conc.	13.4 mill./mL	1	15	7,064,674
L2	Low sperm conc.	15 mill./mL	1	16	8,091,871
L3	Low sperm conc.	3.4 mill./mL	1	23	9,558,667
L4	Low sperm conc.	9.2 mill./mL	2	15	6,148,043
L5	Low sperm conc.	9.7 mill./mL	2	16	4,333,736
L6	Low sperm conc.	5 mill./mL	2	22	6,123,967
L7	Low sperm conc.	1.7 mill./mL	2	25	8,784,711
M1	Medium sperm conc.	38 mill./mL	1	5	7,346,572
M2	Medium sperm conc.	22 mill./mL	1	12	6,260,922
M3	Medium sperm conc.	23 mill./mL	1	14	5,976,608
M4	Medium sperm conc.	36 mill./mL	2	9	4,393,984
M5	Medium sperm conc.	55 mill./mL	2	10	10,054,901
M6	Medium sperm conc.	41 mill./mL	2	27	3,817,299
H1	High sperm conc.	110 mill./mL	1	13	6,461,267
H2	High sperm conc.	127 mill./mL	1	19	5,617,401
H3	High sperm conc.	121 mill./mL	1	20	6,522,668
H4	High sperm conc.	145 mill./mL	2	13	6,334,582
H5 *	High sperm conc.	115 mill./mL	2	14	4,762,306
H6	High sperm conc.	166 mill./mL	2	21	5,564,816
G1	GCNIS	GCNIS	1	6	1,924,818
G2	GCNIS	GCNIS	1	22	8,499,015
G3	GCNIS	GCNIS	1	25	30,972,666
G4	GCNIS	GCNIS	2	7	2,166,453
G5	GCNIS	GCNIS	2	23	10,127,381
NS1	Non-Seminoma	EC	1	2	6,447,654
NS2	Non-Seminoma	EC, S and YST	1	7	4,518,334
NS3	Non-Seminoma	Unknown NS	1	8	9,098,448
NS4	Non-Seminoma	Unknown NS	1	18	7,998,662
NS5	Non-Seminoma	EC, T, S, CHC and YST	1	27	10,326,884
NS6	Non-Seminoma	Unknown NS	2	4	5,249,774
NS7	Non-Seminoma	EC and T	2	6	8,180,864
NS8 *	Non-Seminoma	EC and S	2	8	5,302,999
NS9	Non-Seminoma	Mixed NS	2	12	349,856
NS10	Non-Seminoma	EC	2	20	7,490,740
S1	Seminoma	Seminoma	1	4	6,494,618
S2 *	Seminoma	Seminoma	1	9	4,800,592
S3	Seminoma	Seminoma	1	11	1,444,440
S4	Seminoma	Seminoma	1	21	7,050,519
S5	Seminoma	Seminoma	2	1	5,277,460
S6	Seminoma	Seminoma	2	5	7,338,222
S7	Seminoma	Seminoma	2	18	10,023,884
S8	Seminoma	Seminoma	2	19	4,732,843

Abbreviations; TC: testicular cancer, GCT: Germ cell tumor, Conc.: concentration, GCNIS: Germ cell neoplasia in situ, TGCT: testicular germ cell tumor, EC: embryonal carcinoma, S: seminoma, YST: Yolk sac tumor, NS: non-seminoma, T: teratoma, CHC: choriocarcinoma. * Excluded in the analysis of human small RNAs.

**Table 2:** Small RNAs identified to be differentially present in seminal plasma from men in the indicated groups.

Small RNA	LogFc	LogCPM	LogRatio	*p*-Value	FDR *q*-Value
TGCT/GCNIS vs. controls					
hsa_piR_020345 ^#^	−2.09	3.61	31.60	1.9 × 10^−8^	0.00039
hsa_piR_018580 ^#^	−2.30	1.75	25.07	5.5 × 10^−7^	0.00564
hsa_piR_005838	−3.08	1.27	23.51	1.2 × 10^−6^	0.00844
hsa-miR-149-3p ^§^	−4.59	7.38	22.26	2.4 × 10^−6^	0.01217
hsa_piR_000976	−6.32	6.32	21.23	4.1 × 10^−6^	0.01669
RNU7-182P	−2.28	1.50	20.65	5.5 × 10^−6^	0.01880
hsa_piR_017221	−2.16	1.99	19.84	8.4 × 10^−6^	0.02457
RNU6-94P	−1.51	3.15	19.07	1.3 × 10^−5^	0.03217
hsa-miR-708-5p ^§^	−2.04	1.76	18.84	1.4 × 10^−5^	0.03231
hsa-miR-6855-5p	1.10	3.80	18.46	1.7 × 10^−5^	0.03555
GCNIS vs. controls					
hsa_piR_009051 ^#,§^	3.88	3.86	28.06	1.2 × 10^−7^	0.00122
RNU4-83P	2.80	1.85	28.02	1.2 × 10^−7^	0.00122
hsa_piR_019521 ^§^	3.39	3.97	24.24	8.5 × 10^−7^	0.00581
SNORA2B	2.03	3.42	23.33	1.4 × 10^−6^	0.00698
RNU6-1059P	3.33	3.81	21.37	3.8 × 10^−6^	0.01549
hsa_piR_010894 ^§^	3.59	2.42	19.70	9.0 × 10^−6^	0.03082
hsa-let-7a-5p ^§^	3.62	1.93	18.67	1.6 × 10^−5^	0.04548
SNORD31	3.79	1.60	18.31	1.9 × 10^−5^	0.04694
SNORD118	2.71	2.21	18.13	2.1 × 10^−5^	0.04694
Low vs. high sperm concentrations					
hsa_piR_002438	−9.71	6.71	24.60	7.1 × 10^−7^	0.01442

^#^ Chosen for validation. ^§^ Found in human sperm according to SpermBase [29].

**Table 3:** Small RNAs found in the random forests analysis to be predictive for either TGCT and GCNIS vs. controls or GCNIS vs. controls.

Rank	Small RNA	Type	Mean Decrease Gini	Mean Decrease Accuracy
TGCT/GCNIS vs. controls				
1	hsa_piR_020345 ^#,^*	piRNA	3.29	0.05
2	hsa-miR-6782-5p ^#^	miRNA	2.69	0.02
3	hsa_piR_006001	piRNA	2.53	0.03
4	hsa_piR_022670	piRNA	2.52	0.04
5	hsa_piR_017414	piRNA	2.21	0.02
6	hsa_piR_017112	piRNA	1.92	0.02
7	RNU2-8P	snRNA	1.83	0.02
8	hsa_piR_007418	piRNA	1.62	0.01
9	RNA5SP236	rRNA	1.58	0.01
10	hsa-miR-6511b-3p	miRNA	1.43	0.01
11	hsa-miR-6796-3p	miRNA	1.37	0.01
GCNIS vs. controls				
1	hsa_piR_020345 ^#,^*	piRNA	1.04	0.02
2	RNU6-328P	snRNA	1.00	0.02
3	hsa_piR_016659 ^§^	piRNA	0.74	0.01
4	hsa-miR-4742-3p	miRNA	0.68	0.01
5	hsa-miR-6757-5p	miRNA	0.63	0.01
6	hsa_piR_003309	piRNA	0.50	0.00
7	hsa-miR-5684	miRNA	0.41	0.00

^#^ Chosen for validation. * Additionally found in differential expression analysis, Table 2. ^§^ Found in human sperm according to SpermBase [29].

## Data Availability

All data and materials generated and analyzed during the current study are available from the corresponding author upon reasonable request.

## Supplementary Materials

The following are available online at https://www.mdpi.com/article/10.3390/cancers13102346/s1, Figure S1: MDS plot before exclusion of samples, Figure S2: Distribution of small RNA types in SP from men of the different groups, Figure S3: Distribution of small RNA types in SP from men of the different groups, Table S1: Validation cohort, Table S2: qPCR assays.
