# Supplementary material for: Small RNAs in Seminal Plasma as Novel Biomarkers for Germ Cell Tumors
Source: Cancers (Basel). 2021 May 13;13(10):2346. doi: 10.3390/cancers13102346 (PMC8152278; doi:10.3390/cancers13102346)
Supplement: Supplementary file 1 [file cancers-13-02346-s001.zip › cancers-1202267-supplementary.pdf]

cancers

MDPI

Article

Small RNAs in Seminal Plasma as Novel Biomarkers for Germ Cell Tumors

Nina Mørup, Rytis Stakaitis, Ieva Golubickaite, Meritxell Riera, Marlene D. Dalgaard, Mikkel H. Schierup, Niels Jørgensen, Gedske Daugaard, Anders Juul, and Kristian Almstrup.

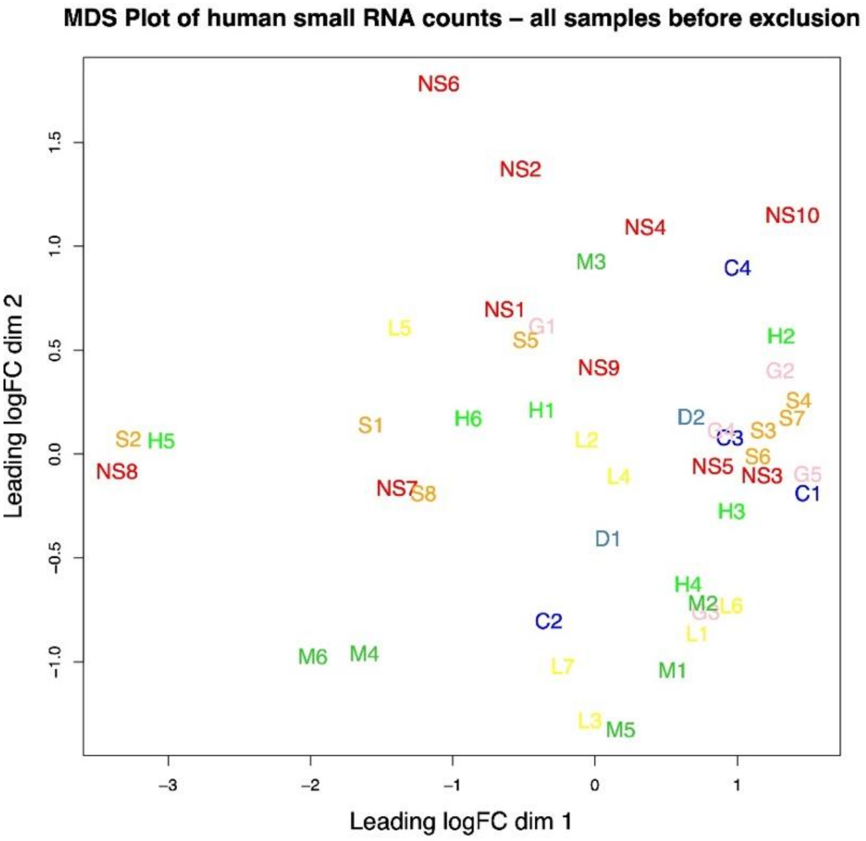

Figure S1. MDS plot before exclusion of samples. Based on the MDS plot samples S2, H5 and NS8 were excluded. Abbreviations; C: cryopreservation control, D: donor, G: GCNIS, H: high sperm count control, L:.low sperm count control, M: medium sperm count control, NS: non-seminoma, and S: seminoma.

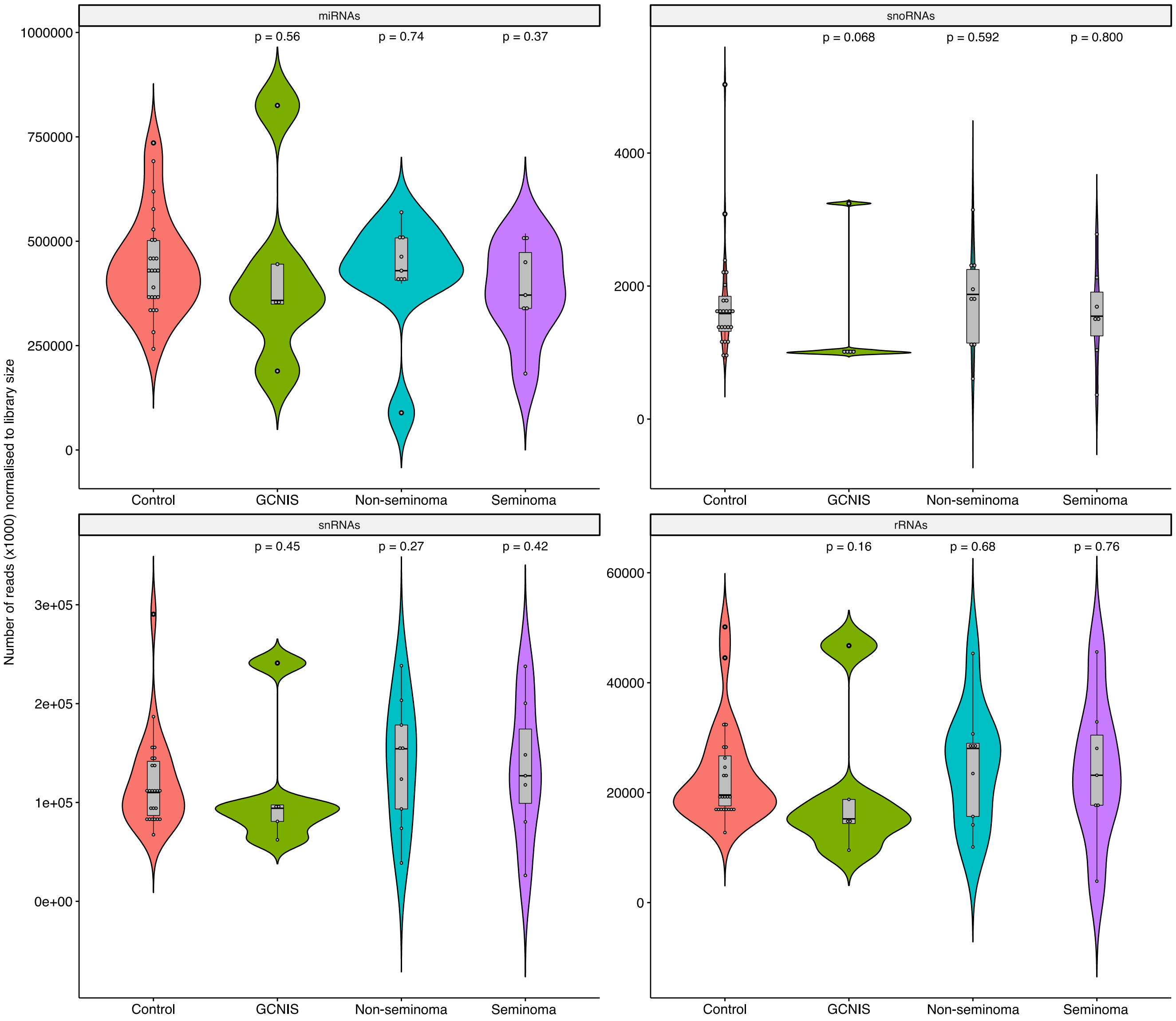

Figure S2. Distribution of small RNA types in SP from men of the different groups.

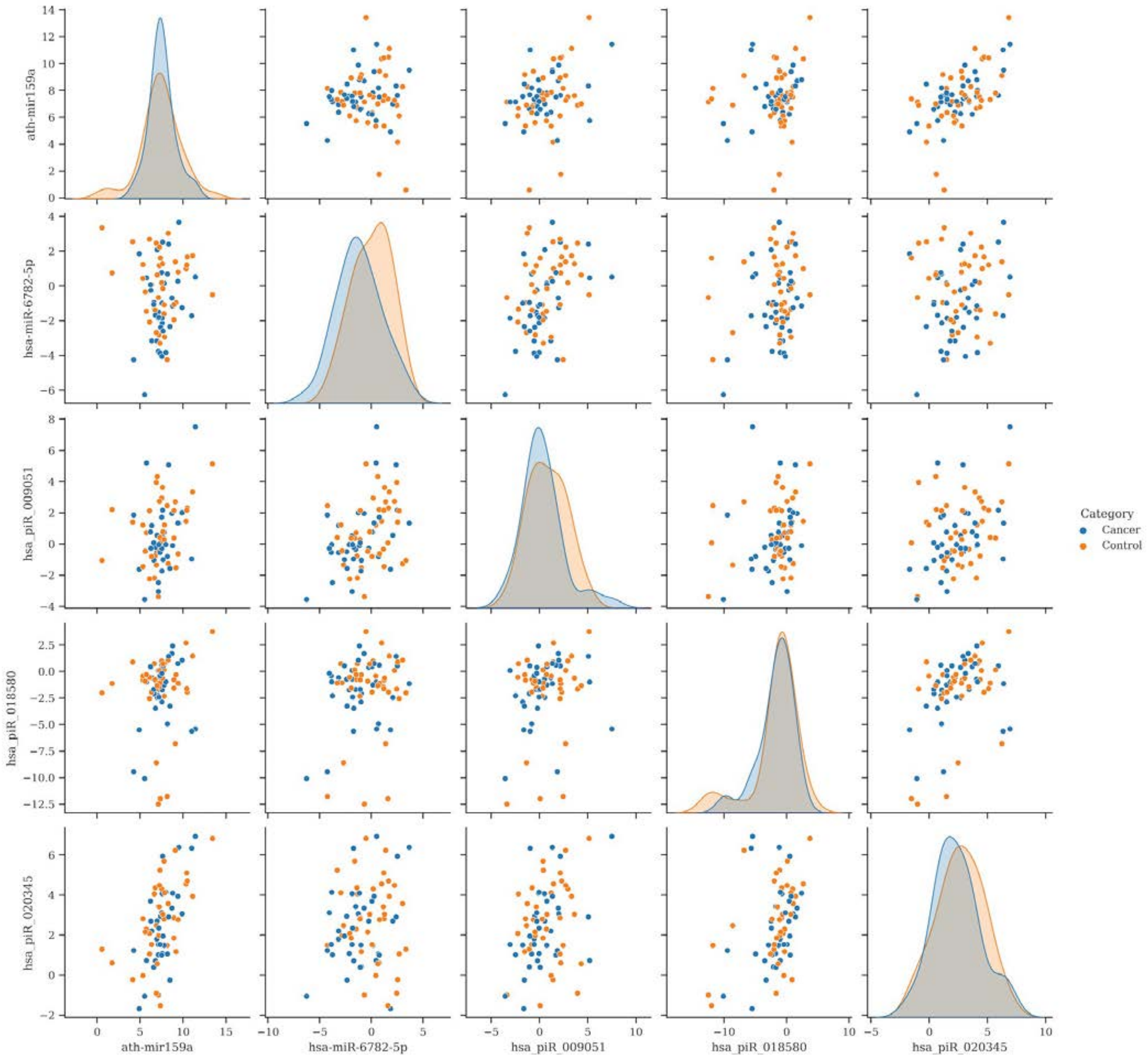

Figure S3. Correlation among seminal plasma small RNA expression from RT-qPCR experiments. Scatter plots showing expression (-ΔCt values) of selected small RNAs. Histogram figures indicate the distribution of expression in TGCT and control groups for each small RNA. Different coloured dots highlight patients from TGCT (blue) and control (orange) groups.

Table S1. Verification cohort, GCNIS/TGCT groups.

Sample ID	Group	Specification	Technical quality
VGCNIS1	GCNIS	GCNIS	Good
VGCNIS2	GCNIS	GCNIS	Good
VGCNIS3	GCNIS	GCNIS	Excluded
VNS1	Non-Seminoma	EC, YST	Good
VNS2	Non-Seminoma	EC, YST, T, CHC	Good
VNS3	Non-Seminoma	EC, YST, T	Good
VNS4	Non-Seminoma	EC, YST, T, CHC	Good
VNS5	Non-Seminoma	EC	Good
VNS6	Non-Seminoma	S, EC, YST, T	Good
VNS7	Non-Seminoma	EC, YST, T	Good
VNS8	Non-Seminoma	EC, T	Good
VNS9	Non-Seminoma	YST, T	Good
VNS10	Non-Seminoma	EC, YST	Good
VNS11	Non-Seminoma	S, EC, YST, T	Good
VNS12	Non-Seminoma	EC, YST, T, CHC	Good
VNS13	Non-Seminoma	EC, YST, T	Good
VNS14	Non-Seminoma	S, EC, YST	Good
VNS15	Non-Seminoma	EC, T	Good
VNS16	Non-Seminoma	EC	Good
VNS17	Non-Seminoma	EC	Good
VNS18	Non-Seminoma	EC, YST, T	Good
VS1	Seminoma	Seminoma	Good
VS2	Seminoma	Seminoma	Good
VS3	Seminoma	Seminoma	Good
VS4	Seminoma	Seminoma	Good
VS5	Seminoma	Seminoma	Good
VS6	Seminoma	Seminoma	Good
VS7	Seminoma	Seminoma	Good
VS8	Seminoma	Seminoma	Good
VS9	Seminoma	Seminoma	Good
VS10	Seminoma	Seminoma	Good
VS11	Seminoma	Seminoma	Good
VS12	Seminoma	Seminoma	Good
VS13	Seminoma	Seminoma	Good
VS14	Seminoma	Seminoma	Good
VS15	Seminoma	Seminoma	Good
VS16	Seminoma	Seminoma	Good
VS17	Seminoma	Seminoma	Good

Abbreviations; GCNIS: Germ cell neoplasia *in situ*, TGCT: testicular germ cell tumor, EC: embryonal carcinoma, S: seminoma, YST: Yolk sac tumor, T: teratoma, CHC: choriocarcinoma.

Table S2. qPCR assays. Assay ordering information for reverse transcription/pre-amplification primers and qPCR TaqMan primers/probes.

Small RNA	Purpose	Design	Target sequence
hsa-miR-6782-5p	Target	Predesigned (466455_mat)	UAGGGGUGGGGGAAUUCAGGGGUGU
hsa_piR_020345	Target	Custom	GGCCAGCCUGGUCCACAUGGGUCGGAA
hsa_piR_009051	Target	Custom	CAGAGUGUAGCUUAACACAAA-GCACCCAACU
hsa_piR_018580	Target	Custom	UUGGUGUUUACGAUGAACAUCGGCAU-GAUGGC
ath-miR159a	Spike-in control	Predesigned (000338)	UUUGGAUUGAAGGGAGCUCUA
hsa-miR-6833-5p	Normalisation control	Predesigned (466277_mat)	GUGUGGAAGAUGGGAGGAGAAA
